# Improved General and Oral Health in Diabetic Patients by an Okinawan-Based Nordic Diet: A Pilot Study

**DOI:** 10.3390/ijms19071949

**Published:** 2018-07-03

**Authors:** Helene Holmer, Cecilia Widén, Viveca Wallin Bengtsson, Michael Coleman, Björn Wohlfart, Stig Steen, Rutger Persson, Klas Sjöberg

**Affiliations:** 1 Kristianstad Central Hospital, SE-29185 Kristianstad, Sweden; Helene.I.Holmer@skane.se; 2 School of Health & Society, Kristianstad University, SE-29188 Kristianstad, Sweden; viveca.wallin_bengtsson@hkr.se (V.W.B.); rutger.persson@hkr.se (R.P.); 3 School of Life and Health Sciences, Aston University, Birmingham B4 7ET, UK; m.d.coleman@aston.ac.uk; 4 Skåne University Hospital, SE-22100 Lund, Sweden; bjorn.wohlfart@med.lu.se (B.W.); stig.steen@med.lu.se (S.S.); 5 Department of Cardiothoracic Surgery, Clinical Sciences Lund, Lund University, SE-22100 Lund, Sweden; 6 Department of Periodontics, School of Dentistry, University of Washington, Seattle, WA 98195, USA; 7 Department of Clinical Sciences, Division of Gastroenterology, Skåne University Hospital, Lund University, SE-20502 Malmö, Sweden; klas.sjoberg@med.lu.se

**Keywords:** diet, oral health, metabolic disorder, cytokines, bleeding on probing, clinical study

## Abstract

Periodontal disease, periodontitis as well as the preceding gingivitis, has been associated with both obesity and diabetes. Studies have shown that diet changes can lead to a lower incidence of such inflammation. The aim of the present case series over four weeks was to study the effects on medical and dental conditions in patients with type 2 diabetes of the consumption of the Okinawan-based Nordic Diet (OBND^®^). Medical and dental examinations were performed to estimate the general health and gingivitis/periodontitis. Serum cytokine levels were assessed using Luminex technology. Eight of ten study participants completed the study. All participants lost weight (*p* = 0.012). Six out of seven that were treated with insulin could reduce their insulin intake after two weeks with OBND^®^. The reduction was about 16 units which corresponds to a 34% relative reduction compared to the starting point (range 15–63%). Fasting blood glucose values fell (*p* = 0.035). Hemoglobin A1c (HbA1c) (*p* = 0.01), triglycerides (*p* = 0.05), and low-density lipoprotein (LDL) (*p* = 0.05) were also reduced. Bleeding on probing changed from ~28% before any dietary changes to ~13% after two weeks with OBND^®^ (*p* = 0.01). The reduction in gingival bleeding was as substantial as might be expected from one session of professional tooth cleaning. Markers of inflammation were also reduced. The OBND^®^ thus showed significant promise in alleviating the impact of diabetes on dental as well as general health.

## 1. Introduction

Obesity poses a major public health challenge worldwide. In the Swedish adult population, approximately 35% is overweight, and with almost 15% defined as being obese [1]. The obesity rate is somewhat higher in southern Sweden [2]. Type 2 diabetes (T2D) is associated with sustained obesity with several health complications. One of the signal complications of both obesity and diabetes is chronic periodontal disease [3]. Periodontitis is a chronic inflammatory disease of the mouth involving the gum tissues (gingiva), the teeth, and the supporting bone. Periodontitis is clinically defined as loss of connective tissue attachment to the teeth, accompanied by alveolar bone loss. Periodontal tissue destruction is caused by host responses to the development of a complex bacterial biofilm [4]. Periodontal infections trigger the release of pro-inflammatory cytokines both at the site of the infection and through the endothelial cell system [5]. Gingivitis is inflammation of the gums and it is generally perceived that periodontitis is preceded by gingivitis, but the etiology of the transition from gingivitis to periodontitis remains unknown.

In the general population, periodontitis is highly prevalent [6]. The Centers for Disease Control and Prevention (CDC, Atlanta, GA, USA) have identified that one out of every two American adults aged 30 and over has periodontitis, and that the prevalence increases to 70% in older adults [6]. In a global review on severe periodontitis, the prevalence in USA (high income) was comparable with that in Western Europe with a prevalence of 7.2% and 9.8%, respectively. The highest numbers were found in Eastern Africa and South America with prevalence rates approaching 20%. In contrast, the lowest prevalence was found in Oceania with only 4% affected [7]. Recent data have identified that dysregulation of biomarkers, with increased glucose, dyslipidemia and hepatic damage can be demonstrated through comorbidity of obesity and periodontitis with effects on both systemic inflammatory and metabolic conditions [8]. Insulin resistance has been associated with both obesity and periodontitis [9,10]. Obesity-induced insulin resistance and vascular gingival inflammation has also been demonstrated [11]. Besides the metabolic syndrome, other variables must be considered when evaluating periodontal parameters, as results could be affected. Professional and domiciliary oral hygiene protocols [12], the presence of orthodontic appliances [13] or the presence of other chronic diseases such as for example chronic obstructive pulmonary disease [14] could be examples of such confounding factors.

Several studies have reported that chronic periodontitis is prevalent in patients with T2D [8,15,16]. The Swedish National Board of Health and Welfare (2015) states in its guidelines for diabetes care that healthcare professionals should refer patients for dental care if patients have ongoing inflammation of the gums (gingivitis or periodontitis). The evidence on the efficacy of routine non-surgical periodontal therapy in individuals with diabetes is contradictory. Some studies have suggested that non-surgical periodontal therapy may be associated with a reduction in hemoglobin A1c (HbA1c) level [17,18]. Other studies have failed to show that non-surgical periodontal therapy or periodontal disease severity were associated with significant changes in serum biomarkers [19,20]. In a Cochrane report, the authors concluded that there is low quality evidence that the treatment of periodontal disease by non-surgical therapy does improve glycemic control in people with diabetes [21].

In poorly controlled T2D, the presence of pro-inflammatory cytokines and chemokines in serum may partially explain the greater susceptibility of T2D patients to periodontal breakdown [22,23]. Fundamental dietary changes may achieve sustainable control of inflammation. The medical benefits of excellent nutrition are well documented and can in many cases result in improved health. Studies have shown that changes in diet can reduce inflammation [24,25], including the control of gingival inflammation [26]. Recent data suggest that a diet low in carbohydrates, rich in omega-3 fatty acids, fibers and vitamins C and D, may significantly reduce gingival and periodontal inflammation [27].

Inhabitants of the Japanese island of Okinawa maintain a traditional low-stress lifestyle, which includes a natural non-refined diet resulting in one of the highest longevity in the world [28]. The typical diet includes a high intake of grains, vegetables, legumes, root vegetables, fish, poultry, fruit and nuts. A modified Okinawan-based Nordic diet (OBND^®^) based on the principle content of the traditional diet on Okinawa has been developed by the Igelösa Life Science AB, Sweden. The diet restricts the intake of sugar, red meat, and dairy products. Data from a recent study included individuals with T2D and used the principles of such a diet; this led to the remarkable finding that several individuals no longer required insulin therapy and others could reduce the daily intake of insulin [29].

The aim of the present pilot study was to study the impact of intake of a specific healthy diet on markers of general and oral health, and markers of inflammation in individuals with T2D. The study more specifically aimed at testing the “proof of principle”, that is, whether adherence to OBND^®^, founded on the recommendations of the Swedish National Food Agency, would result in reductions/changes of clinical and medical/dental parameters of value for the treatment of T2D and gingivitis/periodontitis. Moreover, the study aimed at determining adequate statistical power to be used in a future, case control study.

## 2. Results

**Figure 1:**
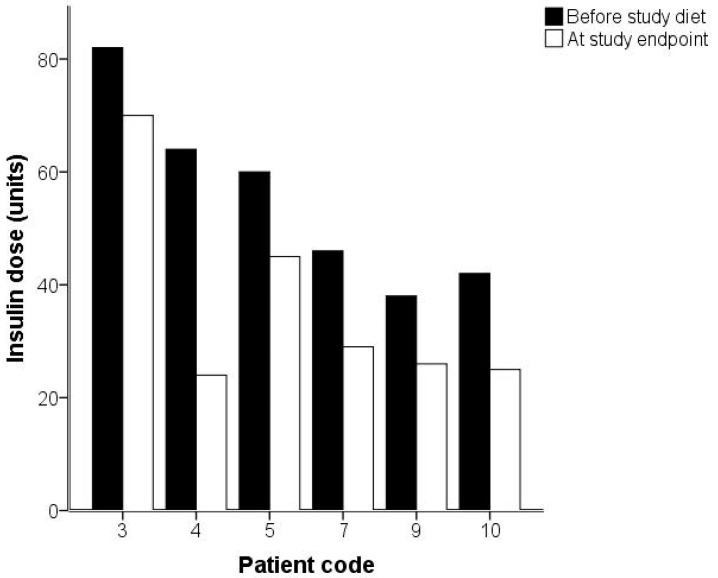
Bar chart diagram illustrating changes in insulin dose for the individuals reducing their insulin treatment after two weeks with the Okinawan-based Nordic diet.

**Figure 2:**
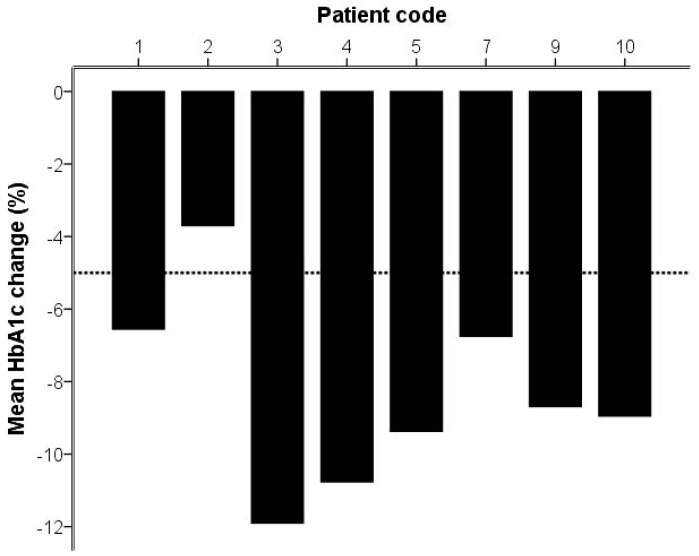
Bar chart diagram illustrating changes in HbA1c for all the participating individuals after two weeks with the Okinawan-based Nordic diet.

Before starting the study diet, one examined individual was admitted to hospital unrelated to study procedures. In addition, one study participant was prescribed antibiotics for a dental procedure and was therefore excluded from the analyses. Thus, eight of the ten enrolled study participants (two females, and six males) diagnosed with T2D completed the study. The mean age of the study group was 59.0 years (S.D. ± 8.1). Descriptive statistics including data on serum variables and general health parameters are presented (Table 1 and Table 2). During the study, all study participants lost weight (*p* = 0.01). Seven of them were under daily treatment with insulin. After two weeks with OBND^®^, 6/7 individuals reduced their insulin intake (Figure 1). The mean reduction was approximately 16 units which corresponds to 34% relative reduction compared to the starting point (range 15–63%; *p* = 0.05). Fasting blood glucose values fell (*p* = 0.05). HbA1c (Figure 2; *p* = 0.01), triglycerides (*p* = 0.05), and low-density lipoprotein (LDL) levels (*p* = 0.05) were also reduced.

### 2.1. Analysis of Gingivitis and Periodontitis

**Figure 3:**
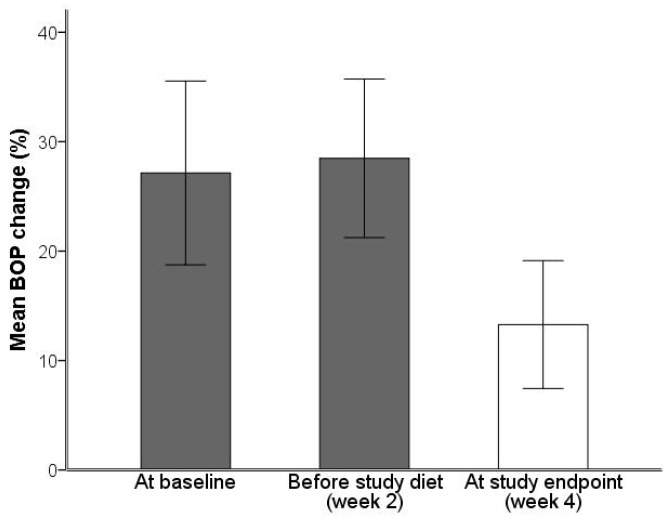
Bar chart diagram illustrating mean changes in bleeding on probing (BOP; %) at different time points of the study (baseline [mean ± S.D.; 27.1 ± 10.0], before study diet [28.5 ± 8.7] and at study endpoint [13.3 ± 7.0]).

At baseline, a diagnosis of gingivitis was identified in 75% of the individuals. Independent *t*-test failed to demonstrate changes in bleeding on probing (BOP) between baseline and 2 weeks (before intake of study diet). Between week 2 and week 4 the reduction in BOP was significant, from ~28% before any dietary changes to ~13% after two weeks with the OBND^®^ (mean diff: 15.2%, standard error (S.E.) diff: 3.9, 95% confidence interval (CI): 23.7, 6.7, *p* = 0.002) (Table 2, Figure 3).

Measurements of panoramic X-rays were performed by a periodontist (RP) without knowledge of medical conditions, age or gender for the study participants. Alveolar bone loss was measured on the panoramic radiographs using a computer software for studies of metric changes (Osiris Pixmeo SARL, Bernex, Switzerland). None of the study individuals showed radiographic evidence of alveolar bone loss suggesting a diagnosis of periodontitis.

### 2.2. Analysis of Gingivitis and Diabetes

All participants reduced their HbA1c levels (*p* = 0.05). Between week 2 and week 4 the reduction in HbA1c was significant, from 68.5 mmol/mol before any dietary changes to 62.5 mmol/mol after two weeks with the OBND^®^ (Table 2). 

**Figure 4:**
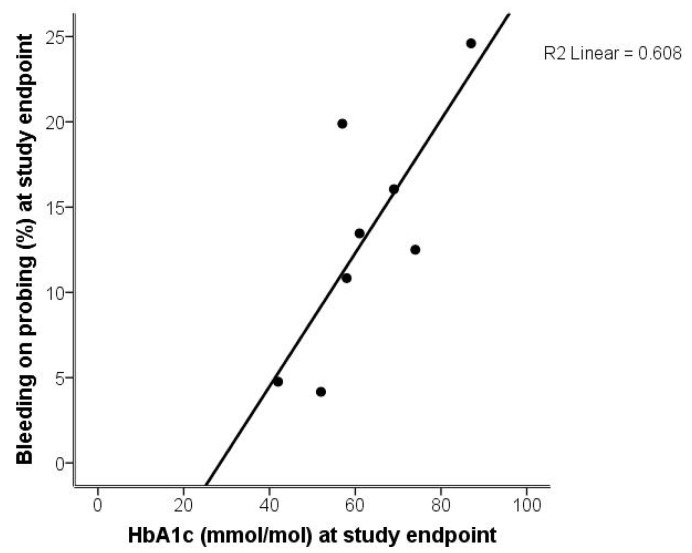
Scatterplot diagram illustrating the relationships between bleeding on probing (BOP; %) and levels of HbA1c (mmol/mol) at study endpoint.

The correlation between BOP and HbA1c at study endpoint are illustrated in a scatterplot diagram (Figure 4).

### 2.3. Analysis of High-Sensitivity C-Reactive Protein and Serum Cytokines

A reduction in high-sensitivity C-reactive protein (hs-CRP) was identified in all individuals but one. Changes in pro-inflammatory cytokines were analyzed in individuals with a reduction of hs-CRP (Table 3). A 10% reduction was identified in five cytokines (interleukin (IL)12, IL13, interferon-inducible protein-10 (IP10, monocyte chemo-attractant protein-1 (MCP)1, and vascular endothelial growth factor (VEGF) and with a 20% only three cytokines remained (IL12, IP10, and MCP1). 6/7 individuals showed a reduction in hs-CRP and 5/7 a reduction in MCP1. The other inflammatory markers did not change to the same extent.

## 3. Discussion

In this 2-week dietary intervention with the Okinawan-based Nordic diet (OBND^®^), significant reductions in weight, body mass index and waist circumference were identified despite the limited number of participants and short intervention period. It should be noted that a clinical reduction in HbA1c levels of 5% is considered good and a satisfactory level for a new pharmaceutical [30,31]. All the participating individuals except one, reduced their HbA1c levels by more than 5%. Furthermore, beneficial effects in glucose and lipid metabolic homeostasis were identified, resulting in reduced insulin intake. This is in line with the previous 12-week study with the OBND^®^ [29]. While any restricted and nutritious diet may have positive benefits, several features make the OBND^®^ unique; (I) the health benefits on clinical parameters were extremely rapid, that is, within two weeks; and (II) the diet has a proven positive impact on the clinical progress of an otherwise debilitating condition such as diabetes. The diet was also well accepted by the study participants. The fact that all participants showed evidence of a substantial comorbidity with metabolic syndrome and diabetic complications should also be considered. Since the inflammatory response is driven by several pro-inflammatory diseases besides diabetes per se the effect of the OBND^®^ diet is probably reduced in this cohort and may be underestimated. 

There are limited data on cost-effectiveness through administration of a well-balanced nutritional diet aimed at individuals with diabetes in relation to insulin requirements, physician care, dental conditions, and overall signs of inflammation. Obesity and T2D are driven by energy-dense diets and sedentary lifestyles [32]. Low-carbohydrate diet has been associated with normal body weight [33]. Okinawa is a Japanese island where the people maintain traditional lifestyle, natural non-refined diet along with low levels of stress, and they are ranked as number one in the world for healthy longevity. The Okinawa diet is characterized by (I) reduced intake of calories; (II) rich sources of antioxidants, and minerals including calcium, iron, potassium; and zinc (III) reduced intake of fat, and sugar; (IV) richness of vegetarian and seafood components. Caloric restriction and traditional Okinawan functional foods may partly explain the extended health and lifespan of the Okinawans [34]. Recent data suggest, however, that life expectancy in Japan is now declining [35]. This may in part be explained by dietary changes and adjustments to similar dietary habits as in other parts of Japan, and worldwide.

A healthy Nordic diet includes intake of fatty fish, low-fat dairy, whole grain cereals such as oats and barley, berries, root vegetables, rapeseed oil, nuts and legumes [36]. Such a diet improves blood lipid profile, insulin sensitivity, and lowers blood pressure and body weight in hyper-cholesterolemic individuals and is associated with decreased morbidity and mortality. An Okinawan-based Nordic diet with moderately low carbohydrate content and high fiber, unsaturated fat, and protein contents renders increased satiety, reduced sweet craving and improved insulin levels, reduction in blood glucose, improvements in body weight, body mass index, total cholesterol, low-density lipoprotein cholesterol, lower systolic and diastolic blood pressure [29]. The health effects are mediated both by certain components in the diet such as fibers, unsaturated fat and antioxidants in vegetables and fruits and by the combined effect of all these factors together. 

Diabetes is a risk factor for increased prevalence of gingivitis and periodontitis approaching almost 70% and 25%, respectively [37]. A survey of periodontal conditions of young adults in Sweden identified that 44% of young adults in Sweden were diagnosed with gingivitis [38]. A diet rich in fruits and vegetables has been associated with better periodontal health as it may reduce gingival bleeding [26] and pocket depths [39]. Although the participating individuals already had relatively good oral hygiene as reflected by gingival inflammation with bleeding on probing approaching 27–28%, this was reduced by more than half after only two weeks of dietary change. In addition to conventional care, that include oral bacteria control [40], in office ultrasonic instrumentation [41] and home care protocols [42] also diet has to be considered a crucial factor for the maintenance of healthy oral conditions. Since periodontitis is an inflammatory condition associated with insulin resistance the fast and vast impact on dental health by the OBND^®^ this kind of intervention should be regarded as an important facet in the treatment regimen of T2D. 

Diabetes and obesity is accompanied by increases in pro-inflammatory cytokine levels. Obesity affects both the innate and the adaptive immune system, which cause negative health impacts on the circulatory system, leading to increased risk of T2D and insulin resistance. This predisposes to higher mortality and morbidity. There is also a link between obesity, insulin resistance and increased adipose tissue inflammation. The present study indicated that predominantly markers of the innate and cell mediated immunity were influenced by the dietary intervention. Adipose tissue produces pro-inflammatory cytokines such as MCP1 by recruiting macrophages into fat [43], thus functioning as a risk marker of atherosclerosis. MCP1 is present at increased concentrations in individuals with T2D [44]. A recently completed study of diet and cytokines identified that a Mediterranean diet resulted in significant reductions of pro-inflammatory biomarkers and MCP1 and macrophage inflammatory protein 1 beta (MIP1β) concentrations in atheroma plaque development as compared to a low-fat diet [45]. Analysis of inflammatory parameters in participating individuals identified that serum concentrations of MCP1 were higher at baseline than after two weeks with the OBND^®^. The dietary change thus provides a change in immune status after only two weeks.

This work is essentially a pilot study with limited data over a shorter time span, although the changes identified herein are clinically encouraging. Another limitation is that the study design is a case series where the participants serve as their own controls though a cross-over design, which does increase the risk for examiner bias during the dental clinical examinations. The short-term duration of the present study is another limitation not allowing to control for compliance and interests in continuing with the OBND^®^. All meals were provided and delivered to the participants with restrictions on supplemental snack intakes. Interviews were made with participants confirming compliance with the diet intake. Furthermore, a family member also received the diet to support compliance. The major limitation of the present pilot study is the lack of control group and lack of long term data. However further studies are needed to confirm the results of this preliminary report with randomized controlled trials.

Based on the above reported studies and available data on blood sugar and lipid values, we met one of the study aims identifying that at least 18 individuals in each group would provide adequate statistical power (α = 0.05, β = 0.85).

The study clearly identified clinically relevant changes in insulin dosage needs and reduced gingival inflammation. In fact, the reduction of gingival inflammation matches improvements following professional dental care. Results from the study show that the potential for dietary-mediated improvement in dental health in vulnerable populations is extremely significant, reducing disease burden and improving quality of life. The impact of this diet in reducing obesity, improving diabetic control, cardiac and dental health, will also have a significant effect in morbidity, and longer life expectancy. Perhaps, dietary changes and implementation of a diet based on the principles of OBND^®^ have potential to be a highly efficient approach to making general improvements in human health world-wide. There is, therefore, a need to strengthen the case for the direct impact of dietary modulation for diabetes prevention and care as well as the management of periodontal conditions. In the next steps prospective randomized controlled trials with a health promoting diet as well as studies aiming at understanding the mechanisms for health benefits on a molecular level are highly warranted.

## 4. Patients and Methods

In compliance with the Declaration of Helsinki, the Regional Ethics Committee in Lund, Sweden, approved the study (Institutional Review Board approval No. 2016/582, 13 September 2016). The study has also been registered (ClinTrials: NCT02916589). All participating individuals signed written informed consent.

The following inclusion criteria for participation were: (I) a medically confirmed diagnosis of T2D in the past 5 or more years; (II) currently 18 years of age or older; (III) having ≥20 remaining teeth, and (IV) from logistic perspectives that study participants were living within a confined area allowing convenient distribution of the daily diets. The exclusion criteria were: (I) treatment with antibiotics during the preceding three months; or (II) no changes in other prescribed medications during the study. Based on information from medical records, potential study participants were identified by an endocrinologist (HH). Identification of potential study individuals were based on the criteria above and elevated serum HbA1c values. 

A total of 12 individuals with T2D were asked to participate in the study. While 2 individuals declined participation due to the amount of time necessary to participate and other logistical issues, 10 individuals were included in the study. As the present report is a pilot study, no sample size has been calculated and the number of patients enrolled was depending on Department flow of works. Medical and dental routine examination procedures were performed two weeks before the change in dietary conditions, immediately before the start of study diet, and after two weeks (study endpoint).

### 4.1. Study Diet

The Okinawan-based Nordic diet was prepared following the principles of the Okinawa diet modified by principles of healthy food in Scandinavia at the kitchen of Igelösa Life Science AB, Lund, Sweden. The study individuals were provided three meals/day including breakfast, lunch, and dinner (Table 4). The diet also included two snacks between meals consisting of a variety of fruits, berries, and seeds. The food was delivered daily at no costs to the participants during the study period.

The meal composition was close to a moderately low carbohydrate rich diet, one of four recommended diets from the Swedish National Food Agency for patients with diabetes [46]. These recommendations were also consistent with international recommendations [47,48]. The diet had a mean calorie intake of approximately 1800 kcal/day.

### 4.2. Medical Examination

All information on diabetes was collected from the available medical records of the study participants. The information included data on serum HbA1c levels, duration of diabetes, diabetic complications, and current insulin intake. Data on general health parameters were collected at baseline and at two, and four weeks; including weight, body mass index (BMI), waist circumference, blood pressure, and heartbeat. Fasting peripheral venous blood samples were drawn at baseline, and at two and four weeks to study changes in serum hemoglobin, leucocytes, thrombocytes, long-term sugar, sodium, potassium, creatinine, cholesterol, triglycerides, high- and low-density lipoproteins, C-reactive protein, aspartate amino transferase (ASAT), alanine amino transferase (ALAT), HbA1c, insulin, S-C-peptide, glucose and a selection of cytokines (see below). 

### 4.3. Periodontal Examination

At baseline, at two weeks, and at four weeks, routine periodontal examinations were performed by a periodontist (VWB). The following recordings were made: probing pocket depths (PPD), bleeding on probing (BOP), and radiographic analysis of the extent of alveolar bone loss (only at the first visit). PPDs at six sites per tooth (PCR-12, Hu-Friedy, Chicago, IL, USA). BOP was assessed 30 s after probing at the sites of pocket probing. BOP was categorized as bleeding or no bleeding. A diagnosis of gingivitis was declared if ≥20% of assessed teeth presented with BOP but in the absence of radiographic evidence of bone loss. A diagnosis of periodontitis was defined as the presence of BOP at ≥20%, and ≥2 sites (not adjacent) with a PPD ≥5 mm, and with ≥10% of sites having radiographic evidence of alveolar bone loss (≥4 mm between cement-enamel junction to alveolar bone) as assessed from panoramic radiographs. Since periodontitis and alveolar bone loss will develop only gradually and over a longer time span gingivitis was the parameter that best could identify improved oral health during the study period. Consequently, this factor was the one that was recorded. 

### 4.4. Cytokine Analysis

A commercially available panel of pro- and anti-inflammatory cytokines was assessed using Luminex MagPix multi-analyte technology (Luminex, Austin, TX, USA). The cytokine analysis was performed according to manufacturer’s instructions for the xMAP technology with multiplex beads. Duplicate readings were made. Fluorescently labelled reporter molecules were measured and cytokine concentrations calculated by Bio-Plex for the following cytokines; Basic FGF (basic fibroblast growth factor), Eotaxin, GCSF (granulocyte colony-stimulating factor), IFN-γ (interferon gamma), Interleukin (IL): IL1β (interleukin 1 beta), IL1ra (receptor antagonist), IL4, IL5, IL6, IL7, IL8, IL9, IL10, IL12p70 (active heterodimer), IL13, IL17A, IP10 (interferon-inducible protein-10), MCP1 (monocyte chemo-attractant protein-1), MIP1α (macrophage inflammatory protein 1 alpha), MIP1β (macrophage inflammatory protein 1 beta), PDGF-BB (platelet-derived growth factor subunit B), TNF-α (tumor necrosis factor-alpha), and VEGF (vascular endothelial growth factor). This set of immune markers provides information on activation of the innate as well as the adaptive immune system including both Th1 (cellular) and Th2 (humoral) immune responses.

### 4.5. Statistical Analysis

The statistical package SPSS 22 for Windows was used for all analyses. Wilcoxon signed-rank test and independent *t*-test were used to assess medical and dental changes before and after intake of study diet. A *p*-value below 0.05 was considered significant. 

## Figures and Tables

**Table 1:** Baseline data of the enrolled patients.

Patient Code	Age (Years)	Gender	Diabetes Duration (Years)	Tobacco	Complications	Medication
1	56	male	6	Quit 2010	Hyperlipidemia, hypertension, myocardial infarction	Metformin
2	72	male	30	Never	Hyperlipidemia, hypertension, retinopathy	Metformin, insulin, Glucagon-like peptide-1
3	51	female	19	Never	Hypertension, sleep apnea syndrome, acromegaly, hyperthyroidism	Levaxin, Genotropin, insulin
4	63	male	24	Quit 1991	Hyperlipidemia, hypertension, retinopathy, nephropathy	Metformin, insulin, Glucagon-like peptide-1
5	65	male	9	Quit 2006	Hyperlipidemia, hypertension, cerebrovascular insult	Insulin
7	63	female	16	Quit 2015	Hyperlipidemia, hypertension	Metformin, insulin, Glucagon-like peptide-1
9	48	male	6	Quit 1990	Hyperlipidemia, hypertension, sleep apnea syndrome	Metformin, insulin
10	54	male	10	Quit 2009	Hypertension, retinopathy, nephropathy, hepatitis C	Insulin

**Table 2:** Mean levels and standard deviations (S.D.) of medical values in individuals with type 2 diabetes (T2D) at 2 weeks (before intake of study diet), and after two weeks with the study diet.

Medical Data	Mean	S.D.	Mean	S.D.	Statistical Significance
	Before Study Diet (Week 2; *n* = 8)		At Study Endpoint (Week 4; *n* = 8)		
Weight (kg)	95.1	11.6	92.3	11.1	*p* = 0.01
Body mass index (BMI; kg/m^2^)	31.8	3.4	30.8	3.4	*p* = 0.01
Waist circumference (cm)	110.6	5.9	107.6	6.6	*p* = 0.01
Systolic blood pressure (mm Hg)	143.5	11.6	138.3	14.9	*p* = 0.26
Diastolic blood pressure (mm Hg)	79.8	8.8	77.8	6.3	*p* = 0.61
Fasting glucose (mmol/L)	10.4	2.7	6.8	2.2	*p* = 0.05
Hemoglobin A1c (HbA1c; mmol/mol)	68.4	16.1	62.5	13.9	*p* = 0.01
Triglycerides (mmol/L)	2.8	1.9	1.5	0.8	*p* = 0.05
Low-density lipoprotein (LDL; mmol/L)	2.5	0.9	1.8	0.8	*p* = 0.05
Bleeding on probing (BOP; %)	28.5	8.7	13.3	7.0	*p* = 0.01

**Table 3:** Median and 25th and 75th percentile of high sensitivity C-reactive protein (hs-CRP) and pro-inflammatory cytokines in individuals with type 2 diabetes at 2 weeks (before intake of study diet), and after two weeks with the study diet. Presentation of cases with either 10% or 20% reduction of pro-inflammatory cytokines.

Variable	Cases with Reduction	Before Study Diet (Week 2)			At Study Endpoint (Week 4)			Significance
		Median	25%	75%	Median	25%	75%	
*≥10% reduction*								
hs-CRP (mg/L)	7	2.3	1.4	2.7	1.6	0.6	2.1	*p* = 0.05
IL12 (pg/mL)	2	54.8	27.1	64.0	37.7	27.4	61.1	NS
IL13 (pg/mL)	5	5.3	4.9	5.3	4.5	4.1	5.3	NS
IP10 (pg/mL)	3	482.3	327.2	776.0	372.6	279.6	915.0	NS
MCP1 (pg/mL)	5	8.2	2.6	10.3	2.1	0.0	9.0	*p* = 0.05
VEGF (pg/mL)	3	182.4	92.8	230.9	157.9	106.3	174.0	NS
*≥20% reduction*								
hs-CRP (mg/L)	6	2.3	1.4	2.7	1.6	0.6	2.1	*p* = 0.05
IL12 (pg/mL)	2	54.8	27.1	64.0	37.7	27.4	61.1	NS
IP10 (pg/mL)	2	482.3	327.2	776.0	372.6	279.6	915.0	NS
MCP1 (pg/mL)	5	8.2	2.6	10.3	2.1	0.0	9.0	*p* = 0.05

**Table 4:** One week of the Okinawan-based Nordic diet.

**Breakfast**						
Porridge or sour milk with muesli, sandwich with cheese or ham, fruit						
**Morning Tea**						
Arranged individually						
**Lunch**						
Day 1	Day 2	Day 3	Day 4	Day 5	Day 6	Day 7
French vegetable soup, basil pesto, wholegrains Apple compote, vanilla yoghurt, Igelösa wholegrain sprinkle	Salmon pudding, dill and mustard yoghurt sauce, cauliflower, cabbage salad with quinoa	Carrot and coconut soup, red lentils and prawns, wholegrains	Salmon with saffron sauce, wholegrains, ratatouille	Italian millet nuggets, basil and sundried tomato yoghurt sauce, grilled vegetables	Sesame fried salmon, black rice, sesame chili sauce, broccoli and carrots	Mush room soup, wholegrains, zucchini bread
**Afternoon Tea**						
Orange and almonds	Pear and bean truffle with taste of orange	Apple and walnuts	Pear and bean truffle with brandy flavoring	Orange and almonds	Apple and a piece of dark chocolate	Pear and walnuts
**Dinner**						
Sausage stroganoff, rice, green peas	Aubergine pizza, cabbage salad with lentils, basil pesto with yoghurt sauce	Grilled chicken, mustard sauce, grilled vegetables Chocolate cake, bilberry, whipped cream	Beef stew, mixed vegetables, wholegrains	Pasta with minced chicken-sauce	Potato burgers with feta cheese and sundried tomatoes, grilled vegetables, tomato cream sauce	Cabbage pudding, brown sauce, lingonberries, grilled vegetables
